# 

**Published:** 2011

Abstract

Pre-exposure prophylaxis (PrEP) for HIV prevention is a promising experimental approach currently being tested globally. A number of PrEP trials are evaluating the safety and effectiveness of PrEP in men who have sex with men (MSM) and other populations at risk for HIV, and results will be available from this first generation of efficacy trials over the next few years. Here we review the rationale for orally-administered antiretrovirals for prevention, and outline issues the first generation trials will address as well as questions that may be addressed in future studies. We also describe the rationale for combination prevention approaches that may combine PrEP with other prevention modalities as part of a larger prevention package.

