# Supplementary material
Source: Int J Prev Med. 2011 Apr-Jun;2(2):105–13. (PMC3093771)
Supplement: Related texts

# Abstract: META-ANALYSIS OF ENDOPHTHALMITIS AFTER INTRAVITREAL INJECTION OF ANTI-VASCULAR ENDOTHELIAL GROWTH FACTOR AGENTS: Causative Organisms and Possible Prevention Strategies

**Authors:** CA McCannel

Abstract

## PURPOSE:

To report the rates of endophthalmitis and the spectrum of causative organisms after intravitreal injection of anti-vascular endothelial growth factor agents and possible prevention strategies.

## METHODS:

Meta-analysis of the US literature from 2005 to 2009 reporting endophthalmitis bacterial isolates after intravitreal injection of anti-vascular endothelial growth factor agents and comparison with reports of endophthalmitis bacterial isolates after intraocular surgery in the United States.

## RESULTS:

Endophthalmitis after intravitreal injection occurred in 52 of 105,536 injections (0.049%) (95% confidence interval [CI], 0.038-0.065%). Among 50 cases of endophthalmitis with bacterial culture isolates, 24 (48.0% [95% CI, 34.8-61.5%]) were culture negative and 26 (52% [95% CI, 38.5-65.2%]) were culture positive. Among the 26 culture-positive isolates, causative organisms were coagulase-negative Staphylococcus in 17 cases (65.4% [95% CI, 46.0-80.6%]), Streptococcus species in 8 cases (30.8% [95% CI, 16.5-50.2%]), and Bacillus cereus in 1 case (3.8% [95% CI, 0.9-19.0%]). Streptococcus species were significantly more frequent after intravitreal injection than after intraocular surgery in the Endophthalmitis Vitrectomy Study (29 of 226 isolates, 9.0% [95% CI, 6.3-12.6%], P = 0.005), a report on clear corneal cataract surgery endophthalmitis (6 of 73 isolates, 8.2% [95% CI, 3.9-16.8%], P = 0.022), and a report on postvitrectomy endophthalmitis with no cases of Streptococcus species.

## CONCLUSION:

Streptococcal isolates are approximately three times more frequent after intravitreal anti-vascular endothelial growth factor injection than after intraocular surgery. Strategies to consider minimizing oropharyngeal droplet transmission may include avoiding talking, coughing, and sneezing or wearing surgical masks.

# Abstract: Safety and effectiveness of BufferGel and 0.5% PRO2000 gel for the prevention of HIV infection in women

**Authors:** SS Abdool Karim, BA Richardson, G Ramjee, IF Hoffman, ZM Chirenje, T Taha, M Kapina, L Maslankowski, A Coletti, A Profy, TR Moench, E Piwowar-Manning, B Mâsse, SL Hillier, L Soto-Torres

Abstract

## OBJECTIVE:

To determine the safety and effectiveness of BufferGel and 0.5% PRO2000 microbicide gels for the prevention of male-to-female HIV transmission.

## DESIGN:

Phase II/IIb, randomized, placebo-controlled trial with three double-blinded gel arms and an open-label no gel arm.

## METHODS:

Study participants from Malawi, South Africa, Zambia, Zimbabwe, and the USA were instructed to apply study gel for 1 h or less before each sex act and safety, sexual behavior, pregnancy, gel adherence, acceptability, and HIV serostatus were assessed during follow-up.

## RESULTS:

The 3101 enrolled women were followed for an average of 20.4 months with 93.6% retention and 81.1% self-reported gel adherence. Adverse event rates were similar in all study arms. HIV incidence rates in the 0.5% PRO2000 gel, BufferGel, placebo gel, and no gel arms were 2.70, 4.14, 3.91, and 4.02 per 100 women-years, respectively. HIV incidence in the 0.5% PRO2000 gel arm was lower than the placebo gel arm (hazard ratio = 0.7, P = 0.10) and the no gel arm (hazard ratio = 0.67, P = 0.06). HIV incidence rates were similar in the BufferGel and both placebo gel (hazard ratio = 1.10, P = 0.63) and no gel control arms (hazard ratio = 1.05, P = 0.78). HIV incidence was similar in the placebo gel and no gel arms (hazard ratio = 0.97, P = 0.89).

## CONCLUSION:

The 0.5% PRO2000 gel demonstrated a modest 30% reduction in HIV acquisition in women. However, these results were not statistically significant and subsequent findings from the Microbicide Development Programme (MDP) 301 trial have confirmed that 0.5% PRO2000 gel has little or no protective effect. BufferGel did not alter the risk of HIV infection. Both products were well tolerated.

# Abstract: Prevalence of sexually transmitted co-infections in people living with HIV/AIDS: systematic review with implications for using HIV treatments for prevention

**Authors:** SC Kalichman, J Pellowski, C Turner

Abstract

Sexually transmitted co-infections increase HIV infectiousness through local inflammatory processes. The prevalence of STI among people living with HIV/AIDS has implications for containing the spread of HIV in general and the effectiveness of HIV treatments for prevention in particular. Here we report a systematic review of STI co-infections in people living with HIV/AIDS. We focus on STI contracted after becoming HIV infected. Electronic database and manual searches located 37 clinical and epidemiological studies of STI that increase HIV infectiousness. Studies of adults living with HIV/AIDS from developed and developing countries reported STI rates for 46 different samples (33 samples had clinical/laboratory confirmed STI). The overall mean point-prevalence for confirmed STI was16.3% (SD=16.4), and median 12.4% STI prevalence in people living with HIV/AIDS. The most common STI studied were Syphilis with median 9.5% prevalence, Gonorrhea 9.5%, Chlamydia 5%, and Trichamoniasis 18.8% prevalence. STI prevalence was greatest at the time of HIV diagnosis, reflecting the role of STI in HIV transmission. Prevalence of STI among individuals receiving HIV treatment was not appreciably different from untreated persons. The prevalence of STI in people infected with HIV suggests that STI co-infections could undermine efforts to use HIV treatments for prevention by increasing genital secretion infectiousness.

# Abstract: Effect of social cognitive theory-based HIV education prevention program among high school students in Nanjing, China

**Authors:** X Li, L Zhang, R Mao, Q Zhao, B Stanton

Abstract

This study was designed to evaluate potential preventive effects of a cultural adaption of the Focus on Kids (FOK) program among Chinese adolescents through a quasi-experimental intervention trial in Nanjing, China. High school students were assigned to either experimental groups (n = 140) or control groups (n = 164) by schools (with three schools in each condition). The participants completed a confidential questionnaire at baseline and 6-month post-intervention with a follow-up rate of 94.4% (287 of 304). The outcome measures included HIV knowledge, HIV-related perceptions based on the protection motivation theory, stigmatizing attitude toward people living with HIV/AIDS (PLWHA), intentions of health-related risk behaviors and sexual intercourse in the previous 6 months. Results showed a significant intervention effect at 6-month post-intervention in increasing HIV knowledge, decreasing perceptions of response cost associated with abstinence and reducing stigmatizing attitudes toward PLWHA, after controlling for key demographic characteristics and relevant baseline measures. Further mediation analysis suggested that HIV knowledge mediated the effect of intervention on stigma reduction. Findings from this study support the feasibility and initial efficacy of the cultural adaptation of FOK HIV prevention program among high school students in China.

# Abstract: Diagnostic, treatment, and prevention protocols for feline heartworm infection in animal sheltering agencies

**Authors:** KF Dunn, JK Levy, KN Colby, RI Michaud

Abstract

Cats are at risk for heartworm infection (Dirofilaria immitis) wherever the disease is endemic in dogs. Diagnosis is more difficult in cats, and little information is available regarding effective palliative and curative treatments for infected cats. In contrast to the challenges of diagnosis and treatment, chemoprophylaxis is highly effective, and current guidelines call for preventive medications to be administered to all cats in endemic areas. The purpose of this study was to survey feline heartworm management protocols used by 400 animal shelters and foster programs in the endemic states of Alabama, Florida, Georgia, and Mississippi. Only 23% of shelters performed feline heartworm testing. The most common reasons for not testing were expense (36%), lack of treatment options for infected cats (18%), and because the agency considers heartworm infections in cats to be less important than in dogs (12%). Most agencies (69%) did not provide preventive medication to cats. Reasons included because testing was not performed (36%), expense (35%), and the perception that local heartworm risk was low (10%). When preventive was provided, feline-labeled broad-spectrum products were used more commonly (81%) than livestock products (14%). The survey also indicated that many policy decisions were based on inaccurate knowledge of feline heartworm prevalence and pathogenesis. Issues of cost, feasibility, and education prevent most Southeastern sheltering agencies from adequately protecting cats against heartworm disease. Practical guidelines tailored to the needs of these agencies should be developed. Subsidized testing and preventive products may facilitate implementation of feline heartworm management protocols in sheltering agencies.

# Abstract: Impact of Comprehensive and Intensive Treatment of Risk Factors Concerning Cardiovascular Mortality in Secondary Prevention: MIRVAS Study

**Authors:** MA Moreno-Palanco, P Ibáñez-Sanz, CC Pablo, A Pizarro-Portillo, F Rodríguez-Salvanés, C Suárez-Fernández

## INTRODUCTION AND OBJECTIVES:

The aim was to determine whether secondary prevention involving the comprehensive and intensive treatment of cardiovascular risk factors reduces cardiovascular events and cardiovascular mortality at 3-year follow up.

## METHODS:

The study design comprised a randomized, controlled, open trial in a routine clinical practice setting. In total, 247 patients who presented with acute coronary syndrome or stroke were selected. They were randomized to comprehensive and intensive treatment of cardiovascular risk factors (n=121) or to follow-up based on usual care (n=126). The main study outcomes were the number of cardiovascular events and cardiovascular mortality at 3-year follow-up. The percentage of patients in whom each risk factor was successfully controlled was a secondary outcome.

## RESULTS:

Overall, 88.8% of patients assigned to the intensive treatment group had a low-density lipoprotein cholesterol level <100mg/dl compared with 56.4% of the usual-care group (relative risk [RR]=1.57; 95% confidence interval [CI], 1.28-1.93), and 75.7% of diabetics had a hemoglobin A(1c) <7% compared with 28.6% of the usual-care group (RR=2.65; 95% CI, 1.13-6.19). There were four deaths due to cardiovascular causes and 26 nonfatal events in the intensive treatment group versus 17 deaths and 54 nonfatal events in the usual-care group. The cumulative survival rate at 3 years was 97.4% in the intervention group and 85.5% in the control group (p=.003).

## CONCLUSIONS:

Secondary prevention involving comprehensive and intensive treatment of cardiovascular risk factors reduced both morbidity and mortality at 3-year follow up.

# Abstract: Canadian Cardiovascular Society Atrial Fibrillation Guidelines 2010: Prevention and Treatment of Atrial Fibrillation Following Cardiac Surgery

**Authors:** LB Mitchell

**Affiliations:** CCS Atrial Fibrillation Guidelines Committee

Abstract

Postoperative atrial fibrillation and atrial flutter (POAF) are the most common complications of cardiac surgery that require intervention or prolong intensive care unit and total hospital stay. For some patients, these tachyarrhythmias have important consequences including patient discomfort/anxiety, hemodynamic deterioration, cognitive impairment, thromboembolic events including stroke, exposure to the risks of antiarrhythmic treatments, longer hospital stay, and increased health care costs. We conclude that prevention of POAF is a worthwhile exercise and recommend that the dominant therapy for this purpose be β-blocker therapy, especially the continuation of β-blocker therapy that is already in place. When β-blocker therapy is contraindicated, amiodarone prophylaxis is recommended. If both of these therapies are contraindicated, therapy with either intravenous magnesium or biatrial pacing is suggested. Patients at high risk of POAF may be considered for first-line amiodarone therapy, first-line sotalol therapy, or combination prophylactic therapy. The treatment of POAF may follow either a rate-control approach (with the dominant therapy being β-blocking drugs) or a rhythm-control approach. Anticoagulation should be considered if persistent POAF lasts >72 hours and at the point of hospital discharge. The ongoing need for any POAF treatment (including anticoagulation) should be reconsidered 6-12 weeks after the surgical procedure.

# Abstract: Canadian Cardiovascular Society Atrial Fibrillation Guidelines 2010: Prevention of Stroke and Systemic Thromboembolism in Atrial Fibrillation and Flutter

**Authors:** JA Cairns, S Connolly, S McMurtry, M Stephenson, M Talajic

**Affiliations:** CCS Atrial Fibrillation Guidelines Committee

Abstract

The stroke rate in atrial fibrillation is 4.5% per year, with death or permanent disability in over half. The risk of stroke varies from under 1% to over 20% per year, related to the risk factors of congestive heart failure, hypertension, age, diabetes, and prior stroke or transient ischemic attack (TIA). Major bleeding with vitamin K antagonists varies from about 1% to over 12% per year and is related to a number of risk factors. The CHADS(2) index and the HAS-BLED score are useful schemata for the prediction of stroke and bleeding risks. Vitamin K antagonists reduce the risk of stroke by 64%, aspirin reduces it by 19%, and vitamin K antagonists reduce the risk of stroke by 39% when directly compared with aspirin. Dabigatran is superior to warfarin for stroke prevention and causes no increase in major bleeding. We recommend that all patients with atrial fibrillation or atrial flutter, whether paroxysmal, persistent, or permanent, should be stratified for the risk of stroke and for the risk of bleeding and that most should receive antithrombotic therapy. We make detailed recommendations as to the preferred agents in various types of patients and for the management of antithrombotic therapies in the common clinical settings of cardioversion, concomitant coronary artery disease, surgical or diagnostic procedures with a risk of major bleeding, and the occurrence of stroke or major bleeding. Alternatives to antithrombotic therapies are briefly discussed.

# Abstract: Cotton textiles modified with citric acid as efficient anti-bacterial agent for prevention of nosocomial infections

**Authors:** S Bischof Vukusic, S Flincec Grgac, A Budimir, S Kalenic

**Affiliations:** Sandra Bischof Vukusic, University of Zagreb, Faculty of Textile Technology, Prilaz baruna Filipovica 28a, 10 000 Zagreb, Croatia, sbischof@ttf.hr

Abstract

Aim. To study the antimicrobial activity of citric acid (CA) and sodium hypophosphite monohydrate (SHP) against gram-positive and gram-negative bacteria, and to determine the influence of conventional and microwave thermal treatments, on the effectiveness of antimicrobial treatment of cotton textiles. Method: Textile material was impregnated with CA and SHP solution and thermally treated by either conventional or microwave drying/curing treatment. Antibacterial effectiveness was tested according to ISO 20743:2009 standard, using absorption method. The surfaces were morphologically observed by scanning electron microscopy, while physical characteristics were determined by wrinkle recovery angles method (DIN 53 891), tensile strength (DIN 53 837), and whiteness degree method (AATCC 110-2000). Results: Cotton fabric treated with CA and SHP showed significant antibacterial activity against MRSA (6.38 log10 treated by conventional drying and 6.46 log10 treated by microwave drying before washing, and 6.90 log10 and 7.86 log10, respectively, after 1 cycle of home domestic laundering washing [HDLW]). Antibacterial activity was also remarkable against S. aureus (4.25 log10 by conventional drying, 4.58 log10 by microwave drying) and against P. aeruginosa (1.93 log10 by conventional drying and 4.66 log10 by microwave drying). Antibacterial activity against P. aeruginosa was higher in samples subjected to microwave than in conventional drying. Antibacterial activity was reduced after 10 HDLW cycles but the compound was still effective. The surface of the untreated cotton polymer was smooth, while minor erosion stripes appeared on the surfaces treated with antimicrobial agent, and long and deep stripes were found on the surface of the washed sample. Conclusion: CA can be used both for the disposable (non-durable) materials (gowns, masks, and cuffs for blood pressure measurement) and the materials that require durability to laundering. The current protocols and initiatives in infection control could be improved by the use of antimicrobial agents applied on cotton carbohydrate polymer.

# Abstract: Recombinant factor VIIa for the prevention and treatment of bleeding in patients without haemophilia

**Authors:** Y Lin, S Stanworth, J Birchall, C Doree, C Hyde

Abstract

## BACKGROUND:

Recombinant factor VIIa (rFVIIa) is licensed for use in patients with haemophilia and inhibitory alloantibodies. It is also increasingly being used for off-license indications to prevent bleeding in operations where blood loss is likely to be high, and/or to stop bleeding that is proving difficult to control by other means.

## OBJECTIVES:

To assess the effectiveness of rFVIIa when used therapeutically to control active bleeding, or prophylactically to prevent (excessive) bleeding in patients without haemophilia.

## SEARCH STRATEGY:

We searched the Cochrane Injuries Group Specialised Register, CENTRAL, MEDLINE, EMBASE and other specialised databases up to 25 February 2009.

## SELECTION CRITERIA:

Randomised controlled trials (RCTs) comparing rFVIIa with placebo, or one dose of rFVIIa with another, in any patient population (except haemophilia). Outcomes were mortality, blood loss or control of bleeding, red cell transfusion requirements, number of patients transfused and thromboembolic adverse events.

## DATA COLLECTION AND ANALYSIS:

Two authors independently assessed potentially relevant studies for inclusion, extracted data and examined risk of bias. We considered prophylactic and therapeutic rFVIIa studies separately.

## MAIN RESULTS:

Twenty-five RCTs were included: 24 were placebo-controlled double-blind RCTs and one compared different doses of rFVIIa. Fourteen trials involving 1137 participants examined the prophylactic use of rFVIIa; 713 received rFVIIa. There was no evidence of mortality benefit (RR 1.06; 95% CI 0.50 to 2.24). There was decreased blood loss (WMD -272 mL; 95% CI -399 to -146) and decreased red cell transfusion requirements (WMD -243 mL; 95% CI -393 to -92) with rFVIIa treatment; however these values were likely overestimated due to the inability to incorporate data from trials showing no difference of rFVIIa treatment compared to placebo. There was a trend in favour of rFVIIa in the number of participants transfused (RR 0.91; 95% CI 0.82 to 1.02). But there was a trend against rFVIIa with respect to thromboembolic adverse events (RR 1.32; 95% CI 0.84 to 2.06).Eleven trials involving 2366 participants examined the therapeutic use of rFVIIa; 1507 received rFVIIa. There were no outcomes where any observed advantage, or disadvantage, of rFVIIa over placebo could not have been observed by chance alone. There was a trend in favour of rFVIIa for reducing mortality (RR 0.89; 95% CI 0.77 to 1.03). However, there was a trend against rFVIIa for increased thromboembolic adverse events (RR 1.21; 95% CI 0.93 to 1.58).

## AUTHORS’ CONCLUSIONS:

The effectiveness of rFVIIa as a more general haemostatic drug, either prophylactically or therapeutically, remains unproven. The use of rFVIIa outside its current licensed indications should be restricted to clinical trials.

# Abstract: Intra-venous fluids for the prevention of severe ovarian hyperstimulation syndrome

**Authors:** MA Youssef, HG Al-Inany, JL Evers, M Aboulghar

Abstract

## BACKGROUND:

Ovarian hyperstimulation syndrome (OHSS) is a serious and potentially fatal complication of ovarian stimulation, which affects 1% to 14% of all in vitro fertilisation (IVF) or intracytoplasmic sperm injection (ICSI) cycles. A number of clinical studies with conflicting results have reported on the use of intravenous fluids such as albumin, hydroxyethyl starch, Haemaccel® and dextran as a possible way for preventing the severe form of OHSS.

## OBJECTIVES:

To review the effectiveness and safety of administration of intravenous fluids such as albumin, hydroxyethyl starch, Haemaccel® and dextran in the prevention of severe ovarian hyperstimulation syndrome (OHSS) in IVF or ICSI treatment cycles.

## SEARCH STRATEGY

We searched the Cochrane Menstrual Disorders and Subfertility Group Specialised Register of controlled trials, the Cochrane Central Register of Controlled Trials (CENTRAL) (The Cochrane Library, to third quarter 2010), MEDLINE (1950 to November 2010), EMBASE (1980 to November 2010) and The National Research Register (to November 2010). The citation lists of relevant publications, review articles, abstracts of scientific meetings and included studies were also searched. The authors were contacted to provide or clarify data that were unclear from the trial reports.

## SELECTION CRITERIA:

Randomised controlled trials (RCTs) which compared the effects of intravenous fluids with placebo or no treatment for the prevention of severe OHSS in high risk women undergoing IVF or ICSI treatment cycles.

## DATA COLLECTION AND ANALYSIS:

Two review authors independently scanned the abstracts, identified relevant papers, assessed inclusion of trials and trial quality and extracted relevant data. Validity was assessed in terms of method of randomisation, allocation concealment and outcomes. Where possible, data were pooled for analysis. A separate analysis of studies was performed for human albumin and hydroxyethyl starch versus placebo or no treatment. Other potential intravenous fluids have been identified, such as Haemaccel and dextran, however no randomised controlled studies on their applicability could be found.

## MAIN RESULTS:

Nine RCTs involving 1660 (human albumin vs placebo) and 487 (HES vs placebo) randomised women, have been included in this review. There was a borderline statistically significant decrease in the incidence of severe OHSS with administration of human albumin (8 RCTs, OR 0.67, 95% CI 0.45 to 0.99).There was a statistically significant decrease in severe OHSS incidence with administration of hydroxyethyl starch (3 RCTs, OR 0.12, 95% CI 0.04 to 0.40). There was no evidence of statistical difference in the pregnancy rate between both groups of treatment.

## AUTHORS’ CONCLUSIONS:

There is limited evidence of benefit from intra-venous albumin administration at the time of oocyte retrieval in the prevention or reduction of the incidence of severe OHSS in high risk women undergoing IVF or ICSI treatment cycles. Hydroxyethyl starch markedly decreases the incidence of severe OHSS.

# Abstract: Prevention of cerebral ischemia-induced memory deficits by inhibition of phosphodiesterase-4 in rats

**Authors:** LX Li, YF Cheng, HB Lin, C Wang, JP Xu, HT Zhang

Abstract

Inhibition of phosphodiesterase-4 (PDE4) by rolipram, a prototypical PDE4 inhibitor, reverses memory impairment produced pharmacologically or genetically. Comparably, much less is known about the effect of rolipram on cerebral ischemia-induced memory deficits. The objective of this study was to determine the effects of rolipram on ischemia-induced memory deficit, neuronal damage, and alteration of PDE4 activity in the hippocampus. Memory was examined using Morris water-maze and step-through passive avoidance tests in rats subjected to global cerebral ischemia with or without repeated treatment with rolipram (0.3 or 1 mg/kg, i.p.); neuronal damage in the hippocampus and PDE4 activity in hippocampal tissues were determined using Nissl staining and HPLC, respectively. In the water-maze test, cerebral ischemia significantly increased the escape latency to reach the platform during acquisition training and decreased the exploration time in the target quadrant in the probe trial test; these were blocked by rolipram in a dose-dependent manner. Rolipram also reduced the distracted platform searches induced by cerebral ischemia. In the passive avoidance test, ischemia decreased the 24-h latency to the dark compartment, which was also blocked by rolipram treatment. In addition, Nissl staining revealed ischemia-induced neuron loss in hippocampal CA1; this was blocked by rolipram. Further, cerebral ischemia led to increases in activity of PDE, primarily PDE4, in the hippocampus, which also was antagonized by rolipram. These results suggest that rolipram prevents cerebral ischemia-induced memory deficits via inhibition of increased PDE4 activity and attenuation of hippocampal, neuronal damages induced by ischemia. PDE4 may be a target for treatment of cognitive disorders associated with cerebral ischemia.

# Abstract: Research Opportunities Related to Establishing Standards for Tobacco Products Under the Family Smoking Prevention and Tobacco Control Act

**Authors:** SS Hecht

## INTRODUCTION:

This paper was written in response to a request from the U.S. National Cancer Institute. The goal is to discuss some research directions related to establishing tobacco product standards under the Family Smoking Prevention and Tobacco Control Act, which empowers the U.S. Food and Drug Administration to regulate tobacco products. Potential research related to tobacco product ingredients, nicotine, and harmful or potentially harmful constituents of tobacco products is discussed. Discussion: Ingredients, which are additives, require less attention than nicotine and harmful or potentially harmful constituents. With respect to nicotine, the threshold level in tobacco products below which dependent users will be able to freely stop using the product if they choose to do so is a very important question. Harmful and potentially harmful constituents include various toxicants and carcinogens. An updated list of 72 carcinogens in cigarette smoke is presented. A crucial question is the appropriate levels of toxicants and carcinogens in tobacco products. The use of carcinogen and toxicant biomarkers to determine these levels is discussed. Conclusions: The need to establish regulatory standards for added ingredients, nicotine, and other tobacco and tobacco smoke constituents leads to many interesting and potentially highly significant research questions, which urgently need to be addressed.

# Abstract: Lifestyle behaviors associated with secondary prevention of coronary heart disease among california adults

**Authors:** A Bellow, JF Epstein, A Parikh-Patel

Abstract

## INTRODUCTION:

Heart disease is the leading cause of death in the United States. People diagnosed with coronary heart disease (CHD) are at an increased risk for illness and death. To reduce this risk, it is recommended that people who are diagnosed with CHD improve their health behaviors. The objective of this study was to assess the prevalence of associated lifestyle risk behaviors among California adults who have CHD.

## METHODS:

From 2005 through 2008, the California Behavioral Risk Factor Surveillance System collected data regarding previous diagnosis of cardiovascular diseases. We used these data to generate descriptive statistics to characterize the risk behaviors among people who had been diagnosed with CHD compared with people who had not, and developed multivariate logistic models to control for confounding variables.

## RESULTS:

Of total respondents, 5% reported previous diagnosis of CHD. Of respondents with CHD, three-quarters were not eating a healthful amount of fruits and vegetables, 66% were overweight or obese, 55% did not engage in regular physical activity, and nearly 15% were smokers. When we controlled for confounding variables, respondents who had been previously diagnosed with CHD were more likely than respondents who had not been diagnosed with CHD to be overweight or obese, to not exercise on a regular basis, and to be current smokers.

## CONCLUSION:

Adults in California with CHD are engaging in behaviors that put them at higher risk of illness and premature death. To lower death rates due to CHD, more public health efforts should target this population.

# Abstract: Efficacy and safety of anticoagulants in the prevention of venous thromboembolism in patients with acute cerebral hemorrhage. a meta-analysis of controlled studies

**Authors:** M Paciaroni, G Agnelli, M Venti, A Alberti, M Acciarresi, V Caso

**Affiliations:** Stroke Unit, Division of Cardiovascular Medicine, University of Perugia, Perugia, Italy

Abstract

The role of anticoagulants for the prevention of Background and Purpose: venous thromboembolism in acute hemorrhagic stroke is uncertain. We performed an updated meta-analysis of studies to obtain the best estimates of the efficacy and safety of anticoagulants for the prevention of venous thromboembolism in Using electronic and manual patients with acute hemorrhagic stroke. Methods: searches of the literature, we identified randomized and non randomized studies comparing anticoagulants (unfractionated heparin or low-molecular-weight heparin or heparinoids) with treatments other than anticoagulants (elastic stockings, intermittent pneumatic compression or placebo) in patients with acute hemorrhagic stroke. Study outcomes included symptomatic and asymptomatic deep venous thrombosis (DVT), symptomatic and asymptomatic pulmonary embolism (PE), any hematoma enlargement or death. Risk Ratio (RR) for individual outcomes were calculated for each study and data from all studies were pooled using the Four studies (2 randomized) involving 1000 Mantel-Haenszel method. Results: patients with acute hemorrhagic stroke met the criteria for inclusion in this meta-analysis. Compared with other treatments, anticoagulants were associated with a significant reduction in PE (1.7% versus 2.9%, RR 0.37, 95% CI: 0.17 to 0.80, p=0.01), a DVT rate of 4.2% compared to 3.3% (RR 0.77, 95% CI: 0.44 to 1.34, p=0.36), an increase in any hematoma enlargement (8.0% versus 4.0%, RR 1.42; 95% CI: 0.57 to 3.53, p=0.45), and a non significant reduction in mortality (16.1% versus 20.9%, RR 0.76; 95% CI: 0.57 to 1.03, p=0.07). Our findings indicate that in patients with hemorrhagic stroke, Conclusions: early anticoagulation is associated with a significant reduction in PE, a non significant reduction in mortality, with the trade-off of a non significant increase in hematoma enlargement. These results must be taken with caution and should encourage the assessment of the clinical benefit of antithrombotic prophylaxis in patients with cerebral bleeding by properly designed clinical trials.

# Abstract: Use of autologous platelet-rich clots for the prevention of local injury bleeding in patients with severe inherited mucocutaneous bleeding disorders

**Authors:** P Nurden, I Youlouz-Marfak, F Siberchicot, E Kostrzewa, I Andia, E Anitua, AT Nurden

Abstract

Stopping or preventing local bleeding in patients with inherited Summary. bleeding disorders linked to abnormal platelet function is traditionally treated by transfusion of blood cell products or recombinant factor VIIa. We now report the use in such patients of autologous platelet-rich clots as an aid to preventing bleeding and to facilitating tissue regeneration at superficial sites. Two patients with von Willebrand’s disease (VWD) type 2B and one patient with type I Glanzmann thrombasthenia were treated after tooth extraction and dental surgery. A fourth patient with platelet-type VWD underwent a skin biopsy. Whereas all four patients had a lifelong history of bleeding complications, the application of an autologous platelet-rich clot immediately after surgery combined with tranexamic acid intake to slow fibrinolysis prevented blood loss and resulted in rapid and normal healing. This new procedure is simple, safe and inexpensive; it provides extra security for patients with a bleeding risk undergoing dentistry or superficial surgery.

# Abstract: Provider Perspectives on Evidence-Based HIV Prevention Interventions: Barriers and Facilitators to Implementation

**Authors:** J Owczarzak, J Dickson-Gomez

Abstract

Abstract Since the beginning of the HIV/AIDS epidemic, community-based organizations (CBOs) have been key players in combating this disease through grassroots prevention programs and close ties to at-risk populations. Increasingly, both funding agencies and public health institutions require that CBOs implement evidence-based HIV prevention interventions, most of which are researcher developed. However, after completing training for these evidence-based interventions (EBIs), agencies may either abandon plans to implement them or significantly modify the intervention. Based on 22 semistructured interviews with HIV prevention service providers, this article explores the barriers and facilitators to dissemination and implementation of EBIs included in the Centers for Disease Control and Prevention’s (CDC) Diffusion of Effective Behavioral Interventions (DEBI) program. Results suggest that there is a tension between the need to implement interventions with fidelity and the lack of guidance on how to adapt the interventions for their constituencies and organizational contexts. Findings suggest the need for HIV prevention intervention development and dissemination that integrate community partners in all phases of research and dissemination.

# Abstract: Vaccines and immunotherapeutics for the prevention and treatment of infections with West Nile virus

**Authors:** DW Beasley

**Affiliations:** Department of Microbiology & Immunology, Sealy Center for Vaccine Development, Center for Biodefense & Emerging Infectious Diseases, Institute for Human Infections & Immunity, &; Galveston National Laboratory, The University of Texas Medical Branch, Galveston, TX 77555-0609, USA. d.beasley@utmb.edu

Abstract

The emergence of West Nile virus (WNV) in North America in 1999 as a cause of severe neurological disease in humans, horses and birds stimulated development of vaccines for human and veterinary use, as well as polyclonal/monoclonal antibodies and other immunomodulating compounds for use as therapeutics. Although disease incidence in North America has declined since the peak epidemics in 2002-2003, the virus has continued to be annually transmitted in the Americas and to cause periodic epidemics in Europe and the Middle East. Continued transmission of the virus with human and animal disease suggests that vaccines and therapeutics for the prevention and treatment of WNV disease could be of great benefit. This article focuses on progress in development and evaluation of vaccines and immunotherapeutics for the prevention and treatment of WNV disease in humans and animals.

# Abstract: Rational care or rationing care? Updates and controversies in women’s prevention

**Authors:** L Davisson

Abstract

Prevention has potential benefits, but the majority of people undergoing disease screening will receive no benefit and may actually be exposed to health risks. Public opinion is generally very favorable toward prevention. However, many recent guidelines recommend fewer preventive services in women than previously suggested. New recommendations are to wait until 50 for mammography screening, to screen only every other year, and to not teach self breast examinations. Papanicolaou tests for cervical cancer screening are recommended to be done less often (every 2-3 years) and to be started later than previously suggested (not before age 21). Screening for ovarian cancer is not recommended. Guidelines suggest avoiding hormone therapy for primary prevention of coronary heart disease, not giving aspirin to prevent myocardial infarctions in women, and not screening women without risk factors for hyperlipidemia. These recommendations have caused confusion and, because of being revealed during a national health reform debate, have even been perceived as “rationing care.” Others see them as “rational care,” because they encourage utilization of beneficial services while discouraging use of those that may lead to more harms than benefits. Development of prevention guidelines requires value judgments, so despite the use of evidence, these recommendations have not all achieved widespread support. Understanding the data behind the guidelines, health care providers can decide how to approach prevention in practice, taking into consideration individual patient risk factors and preferences.

# Abstract: Complementary and alternative medicine for prevention and treatment of the common cold

**Authors:** R Nahas, A Balla

**Affiliations:** Seekers Centre for Integrative Medicine, Ottawa, ON. richard@seekerscentre.com

Abstract

## OBJECTIVE:

To review the evidence supporting complementary and alternative medicine approaches to treatment and prevention of the common cold in adults.

## QUALITY OF EVIDENCE:

MEDLINE, EMBASE, and the Cochrane Database of Systematic Reviews were searched from January 1966 to September 2009 combining the key words common cold or influenza with echinacea, garlic, ginseng, probiotics, vitamin C, and zinc. Clinical trials and prospective studies were included.

## MAIN MESSAGE:

For prevention, vitamin C demonstrated benefit in a large meta-analysis, with possibly increased benefit in patients subjected to cold stress. There is inconsistent evidence for Asian ginseng (Panax ginseng) and North American ginseng (Panax quinquefolius). Allicin was highly effective in 1 small trial. For treatment, Echinacea purpurea is the most consistently useful variety; it was effective in 5 of 6 trials. Zinc lozenges were effective in 5 of 9 trials, likely owing to dose and formulation issues. Overall, the evidence suggests no benefit from probiotics for prevention or treatment of the common cold.

## CONCLUSION:

Vitamin C can be recommended to Canadian patients for prevention of the common cold. There is moderate evidence supporting the use of Echinacea purpurea and zinc lozenges for treatment. Ginseng and allicin warrant further research.

# Abstract: Dysfunction of the Fusion of Pre-Synaptic Plasma Membranes and Synaptic Vesicles Caused by Oxidative Stress, and its Prevention by Vitamin E

**Authors:** M Arai, M Saito, H Takatsu, K Fukui, S Urano

Abstract

To define whether hyperoxia induces the dysfunction of membrane fusion between synaptic vesicles with pre-synaptic plasma membranes in the nerve terminals, and whether vitamin E prevents this abnormal event, we investigated the influence of hyperoxia on the fusion ability of isolated synaptic vesicles and the inside-out type pre-synaptic plasma membrane vesicles from rat brain using the fluorescence tracing method. The membrane fusion ability of both membranes from rats subjected to hyperoxia was markedly decreased compared with the membranes from a normal rat. Rats subjected to hyperoxia in the form of oxidative stress showed significant increases in the levels of thiobarbituric acid reactive substances (TBARS), conjugated dienes, and protein carbonyl moieties in both synaptic vesicles and pre-synaptic plasma membranes. When rats were supplemented with vitamin E, these abnormalities were inhibited even when rats were subjected to hyperoxia.

# Abstract: T(H)17-Based Vaccine Design for Prevention of Streptococcus pneumoniae Colonization

**Authors:** KL Moffitt, TM Gierahn, YJ Lu, P Gouveia, M Alderson, JB Flechtner, DE Higgins, R Malley

Abstract

Streptococcus pneumoniae is a leading cause of mortality in young children. While successful conjugate polysaccharide vaccines exist, a less expensive serotype-independent protein-based pneumococcal vaccine offers a major advancement for preventing life-threatening pneumococcal infections, particularly in developing nations. IL-17A-secreting CD4+ T cells (T(H)17) mediate resistance to mucosal colonization by multiple pathogens including S. pneumoniae. Screening an expression library containing >96% of predicted pneumococcal proteins, we identified antigens recognized by T(H)17 cells from mice immune to pneumococcal colonization. The identified antigens also elicited IL-17A secretion from colonized mouse splenocytes and human PBMCs suggesting that similar responses are primed during natural exposure. Immunization of two mouse strains with identified antigens provided protection from pneumococcal colonization that was significantly diminished in animals treated with blocking CD4 or IL-17A antibodies. This work demonstrates the potential of proteomic screening approaches to identify specific antigens for the design of subunit vaccines against mucosal pathogens via harnessingT(H)17-mediated immunity.

# Abstract: Treatment and prevention of cryptosporidiosis: what options are there for a country like Zambia?

**Authors:** P Kelly

Abstract

Summary Cryptosporidiosis is a major infection of humans, leading to diarrhoea and growth failure in children, diarrhoea and malnutrition in immunocompromised adults, and is associated with increased mortality in all age groups. Using the country of Zambia as an example, I review the possible approaches to treatment and prevention in a tropical setting. The current optimal therapy for cryptosporidiosis is nitazoxanide which works well in HIV uninfected children, but treatment in patients with HIV infection remains remarkably difficult. No single drug has demonstrated efficacy in a randomised trial. No vaccine is available, so the best option for prevention for the moment is filtration and clean storage of drinking water. This would be expected to reduce cryptosporidiosis dramatically, but this needs to be demonstrated directly. Water filtration would have the added benefit of protection against many other pathogens, but the paucity of alternative approaches highlights the need for a better understanding of this important human pathogen

# Abstract: Evaluation of tenoxicam on prevention of arachnoiditis in rat laminectomy model

**Authors:** B Cemil, G Kurt, C Aydin, N Akyurek, B Erdogan, N Ceviker

Abstract

Post laminectomy arachnoiditis has been shown by experiments with rats and post operative radiological imaging in humans. The purpose of this experimental study was to determine the efficacy of tenoxicam in preventing arachnoiditis in rats. Twenty-four Wistar rats were divided into two groups, and L3 laminectomy was performed. In the tenoxicam group, 0.5 mg/kg tenoxicam was applied intraperitoneally. Normal saline was applied intraperitoneally in the control group. Later, the rats were killed at weeks 3 and 6, and the laminectomy sites were evaluated pathologically for arachnoiditis. The results showed that 6 weeks after surgery, the tenoxicam group showed lowest arachnoiditis grades. However, statistically significant difference was not found in arachnoiditis between the control group and the tenoxicam group. Based on these findings it is concluded that application of the tenoxicam after lumbar laminectomy did not effectively reduce arachnoiditis. Performing the most effective surgical technique without damage around tissue in a small surgical wound and having meticulous hemostasis in surgery seem to be the key for preventing arachnoiditis effectively.

# Abstract: Cost-utility of aspirin and proton pump inhibitors for primary prevention

**Authors:** SR Earnshaw, J Scheiman, AM Fendrick, C McDade, M Pignone

Abstract

## BACKGROUND:

Aspirin reduces myocardial infarction but increases gastrointestinal tract (GI) bleeding. Proton pump inhibitors (PPIs) may reduce upper GI bleeding. We estimate the cost-utility of aspirin treatment with or without a PPI for coronary heart disease (CHD) prevention among men at different risks for CHD and GI bleeding.

## METHODS:

We updated a Markov model to compare costs and outcomes of low-dose aspirin plus PPI (omeprazole, 20 mg/d), low-dose aspirin alone, or no treatment for CHD prevention. We performed lifetime analyses in men with different risks for cardiovascular events and GI bleeding. Aspirin reduced nonfatal myocardial infarction by 30%, increased total stroke by 6%, and increased GI bleeding risk 2-fold. Adding a PPI reduced upper GI bleeding by 80%. Annual aspirin cost was $13.99; the generic PPI cost was $200.00.

## RESULTS:

In 45-year-old men with a 10-year CHD risk of 10% and 0.8 per 1000 annual GI bleeding risk, aspirin ($17 571 and 18.67 quality-adjusted life-years [QALYs]) was more effective and less costly than no treatment ($18 483 and 18.44 QALYs). Compared with aspirin alone, aspirin plus PPI ($21 037 and 18.68 QALYs) had an incremental cost per QALY of $447 077. Results were similar in 55- and 65-year-old men. The incremental cost per QALY of adding a PPI was less than $50 000 per QALY at annual GI bleeding probabilities greater than 4 to 6 per 1000.

## CONCLUSIONS:

Treatment with aspirin for CHD prevention is less costly and more effective than no treatment in men older than 45 years with greater than 10-year, 10% CHD risks. Adding a PPI is not cost-effective for men with average GI bleeding risk but may be cost-effective for selected men at increased risk for GI bleeding
